# AIF-1, a potential biomarker of aggressive tumor behavior in patients with non-small cell lung cancer

**DOI:** 10.1371/journal.pone.0279211

**Published:** 2022-12-15

**Authors:** Lingling Wang, Xing Zhao, Huachuan Zheng, Cuimin Zhu, Yanhong Liu

**Affiliations:** 1 Department of Laboratory Diagnosis, The Second Affiliated Hospital of Harbin Medical University, Harbin, China; 2 Department of Laboratory Diagnosis, The Affiliated Hospital of Chengde Medical University, Chengde, China; 3 Department of Pathology, The Affiliated Hospital of Chengde Medical University, Chengde, China; 4 Department of Oncology and Experimental Center, The Affiliated Hospital of Chengde Medical University, Chengde, China; 5 Department of Oncology, The Affiliated Hospital of Chengde Medical University, Chengde, China; Inha University Hospital, REPUBLIC OF KOREA

## Abstract

Allogeneic inflammatory factor-1 (AIF-1) overexpression has been reported to be associated with tumorigenesis and tumor metastasis. This study aimed to investigate the role of AIF-1 in the development and progression of non-small cell lung cancer (NSCLC). AIF-1, IL-6, and VEGF expressions in human NSCLC tissue were examined by immunofluorescence staining. Bioinformatics analyses were performed to identify AIF-1-related molecules and pathways in NSCLC. Human lung cancer A549 cell proliferation was assessed by CCK-8 assay, and cell migration was evaluated with wound-healing assay. IL-6 and VEGF secretions in A549 cell culture supernatants were quantified using the Elecsys IL-6 immunoassay kit and Vascular Endothelial Growth Factor Assay Kit. RT-PCR and western blot were performed to quantify the expressions of AIF-1, IL-6, and VEGF mRNAs and proteins involved in p38-MAPK and JAK/STAT3 signaling such as p-p38 and p-STAT3. The effects of AIF-1 on A549 cell proliferation and the expressions of IL-6 and VEGF were assessed using SB203580 and ruxolitinib. The results showed that AIF-1 expression was higher in human NSCLC tissue than that in paracancer tissue. High AIF-1 expression was associated with metastasis, higher TNM stage, and poorer survival. Bioinformatics connected AIF-1 to JAK/STAT signaling in NSCLC. AIF-1 increased A549 cell proliferation, migration, IL-6 secretion and, VEGF secretion, and these effects were attenuated by inhibition of p38-MAPK or JAK/STAT3 signaling. In conclusion, AIF-1 may promote aggressive NSCLC behavior via activation of p38-MAPK and JAK/STAT signaling.

## Introduction

Lung cancer is the main cause of tumor-related deaths worldwide [1]. Globally, there were about 228,820 new cases of lung cancer and 135,720 deaths due to lung cancer in 2020 [2]. Furthermore, there were 815,563 new cases of lung cancer and 714,699 lung cancer-related deaths in China in the same year [3]. Non-small cell lung cancer (NSCLC) accounts for more than 80% of lung cancer cases and includes adenocarcinoma, squamous cell carcinoma (SCC) and large cell carcinoma [4]. Risk factors for lung cancer include family history of lung cancer, smoking, air pollution, dietary factors and occupational exposures [5]. The main treatments for NSCLC are surgery, radiotherapy and chemotherapy, but the overall 5-year survival rate is only 25% [6].

Inflammation plays a role in tumor formation and development through a variety of mechanisms including epigenetic changes that alter gene expression, vascular abnormalities and inflammatory mediator release [7,8]. There is evidence that chronic inflammation caused by smoking, bronchial asthma or long-term inhalation of silica dust can induce lung cancer [9]. Various cytokines and growth factors have been implicated in lung cancer. Interleukin-6 (IL-6) can promote the migration, invasion and metastasis of NSCLC cells [10,11] and enhance the resistance of NSCLC cells to chemotherapy [12]. Furthermore, elevated serum IL-6 levels are associated with a poor prognosis in patients with NSCLC [13]. Vascular endothelial growth factor (VEGF) is an angiogenic factor thought to play an important role in the pathogenesis of NSCLC. Like IL-6, VEGF stimulates the migration and invasion of NSCLC cells [14], and VEGF expression in NSCLC is associated with more advanced TNM stage, dedifferentiation, lymph-node involvement and poorer prognosis [15]. Inhibition of VEGF-A with bevacizumab was found to improve progression-free survival in patients with NSCLC treated with erlotinib (an epidermal growth factor receptor tyrosine kinase inhibitor) [16]. Thus, activation of IL-6- and VEGF-dependent signaling plays an important role in NSCLC progression.

Allograft inflammatory factor-1 (AIF-1) is a 17-kD calcium-binding protein with an EF-hand domain that was first reported in rat cardiac allografts undergoing chronic rejection [17]. AIF-1 was expressed in macrophages, monocytes and neutrophils and up-regulated by interferon-gamma (IFN-γ), suggesting that it may play a role in the function of macrophages [17]. AIF-1 augments macrophage phagocytic activity and accelerates atherosclerosis in apolipoprotein E-deficient mice [18] and enhances the proliferation of vascular smooth muscle cells (VSMCs) through cell cycle regulation [19]. AIF-1 is thought to promote inflammation, antigen presentation and T cell polarization [20,21], and AIF-1 overexpression was observed to stimulate IL-6, IL-10 and IL-12 production by a mouse macrophage cell line [22]. Interestingly, high AIF-1 expression is associated with numerous diseases including rheumatoid arthritis [23], endometriosis [24], systemic sclerosis [25], arteriosclerosis [26], diabetic nephropathy [27] and tumors [28,29]. However, whether AIF-1 plays a role in NSCLC remains unknown.

The aim of this study was to explore whether AIF-1 may play a role in the development of NSCLC. To achieve this objective, we investigated whether AIF-1 expression in tumor tissue samples from patients with NSCLC was associated with CD68 expression (a marker of monocytes such as macrophages), IL-6 expression, VEGF expression and clinicopathological features of aggressive tumor behavior. Additionally, the effects of AIF-1 expression on the proliferation and migration of human lung adenocarcinoma cells and their secretion of IL-6 and VEGF were assessed, and the underlying mechanisms were investigated. It was envisaged that the study findings would provide new insights into the possible role of AIF-1 in NSCLC and provide preliminary information as to whether AIF-1 might be a candidate therapeutic target.

## Materials and methods

### Study participants

NSCLC and paired paracancer surgical tissue specimens (*n* = 47) were obtained from the department of pathology, Affiliated Hospital of Chengde Medical University, China between January 2021 and October 2021. The mean age of the 47 participants (28 men and 19 women) was 60 years-old (age range, 39–76 years old), and there were 21 cases with lymph node metastasis. No cases had distant metastasis. No patient had undergone treatment before the surgical excision of tumor tissue(Table 1). Clinicopathological data including TNM stage [30] were obtained. The Ethics Committee of the Affiliated Hospital of Chengde Medical University approved the study protocol (approval number: CYFYLL2021169). All patients provided informed written consent for their tissue samples to be used in this research. We had not access to information that could identify individual participants during or after data collection.

**Table 1:** Clinical characteristics of patients.

Characteristics	AIF-1 high group (n = 30)	AIF-1 low group (n = 17)	*X* ^2^	P value
Age (years)			0.949	0.330
< 60	15 (50.0)	6 (35.3)		
≥ 60	15 (50.0)	11 (64.7)		
Gender(%)			0.487	0.485
Male	19 (63.3)	9 (52.9)		
Female	11 (36.7)	8 (47.1)		
Smoking(%)			2.884	0.089
Yes	20 (66.7)	7 (41.2)		
No	10 (33.3)	10 (58.8)		
Drinking(%)			0.006	0.937
Yes	12 (40.0)	7 (41.2)		
No	18 (60.0)	10 (58.8)		
High blood pressure(%)			0.206	0.650
Yes	5 (16.7)	2 (11.8)		
No	25 (83.3)	15 (88.2)		
Diabetes(%)			0.024	0.877
Yes	4 (13.3)	2 (11.8)		
No	26 (86.7)	15 (88.2)		
Dyslipidemia(%)			0.774	0.379
Yes	4 (20.0)	1 (5.9)		
No	24 (80.0)	16 (94.1)		
ECOG PS(%)			1.498	0.595
0	12 (40.0)	10 (58.8)		
1	13 (43.3)	5 (29.4)		
2	5 (16.7)	2 (11.8)		
Initial clinical stage(%)			6.992	0.092
ⅠA	14 (46.7)	13 (76.5)		
ⅠB	0 (0.0)	1 (5.9)		
ⅡB	5 (16.7)	2 (11.8)		
ⅢA	8 (26.7)	1 (5.9)		
ⅢB	3 (10.0)	0 (0.0)		

### Histology

Tissue sections were dewaxed with xylene and rehydrated in a graded series of alcohols. The histological characteristics of the tissue samples were determined after staining with hematoxylin and eosin (HE) using standard techniques.

### Immunohistochemistry

The tissue sections were dewaxed with xylene and rehydrated in a graded series of alcohols. High-pressure antigen repair with citrate was performed using a microwave oven. The tissue sections were placed in anhydrous methanol containing 0.3% H_2_O_2_ for 15 minutes to block endogenous peroxidase activity and then incubated with 1% bovine serum albumin (BSA). Subsequently, the tissue sections were incubated with primary antibodies overnight at 4°C followed by horseradish peroxidase (HRP)-conjugated secondary antibodies (1:200; Dako, Agilent Technologies, Santa Clara, CA, USA) for 60 minutes at room temperature. Diaminobenzidine (DAB) was used as the chromogen. Next, the sections were stained with hematoxylin, dehydrated and fixed. The primary antibody was omitted in negative controls. Two independent observers evaluated the nuclear expression of Ki67 and p53 in 100 randomly selected cells within a representative region of the section in a blinded manner (BX53 microscope; Olympus, Tokyo, Japan). Expression was graded as negative (nuclear staining in < 5% of cells), weakly positive (6%–25%), moderately positive (26%–50%) or strongly positive (> 50%).

### Immunofluorescence staining

The tissue sections were dewaxed with xylene and rehydrated in a graded series of alcohols. Antigen repair was carried out by incubation with citrate buffer at 100°C for 15 minutes, and the sections were incubated with 1% BSA for 20 minutes at 37°C to block non-specific binding. The sections were incubated with primary antibodies (1:100; S1 Table) overnight at 4°C and then treated with fluorescein isothiocyanate (FITC)-conjugated secondary antibody (1:200; Wanleibio, Shenyang, China) for 90 minutes at room temperature. The nuclei were counterstained with 4’,6-diamidino-2-phenylindole (DAPI; Sigma-Aldrich, St. Louis, MO, USA). Tissue sections stained without primary antibody were used as negative controls. The sections were observed using a BX60 microscope (Olympus, Tokyo, Japan).

### Immunofluorescence double-labeling

Double-staining of formalin-fixed, paraffin-embedded tissue sections was performed after antigen retrieval in citrate buffer and incubation with 1% BSA to block non-specific binding of immunoglobulins. First, the sections were incubated with mouse anti-CD68 primary antibody (Santa Cruz Biotechnology, Santa Cruz, CA, USA) and rabbit anti-AIF-1 primary antibody (Wanleibio, Shenyang, China) overnight at 4°C. Next, the sections were treated with tetramethylrhodamine-conjugated goat anti-mouse IgG antibody (1:200; SeraCare, Milford, MA, USA) and FITC-conjugated goat anti-rabbit IgG antibody (1:200; Wanleibio, Shenyang, China) for 90 minutes. The nuclei were counterstained with DAPI. The sections were observed using a BX60 microscope (Olympus, Tokyo, Japan).

### Bioinformatics analysis

Data for lung adenocarcinoma and lung SCC were downloaded from The Cancer Genome Atlas (TCGA) database using the TCGA-assembler of R software (R Core Team, Vienna, Austria). The correlations between CD68, IL6, VEGF, and AIF-1 were analyzed using the data from TCGA. Kaplan–Meier plots and TCGA-assembler were employed to analyze the prognostic significance of AIF-1 mRNA in NSCLC. Analyses of AIF-1-related molecules and GO_KEGG pathway enrichment were performed using R software. Results were analyzed using the Ualcan interactive web resource (ualcan.path.uab.edu/home) and Xiantao platform (www.xiantao.love/products).

### Cell culture

The human lung cancer A549 cell line was kindly provided by Prof. Zhao, Respiratory Department, The Affiliated Hospital of Chengde Medical University, China. The cells were cultured in RPMI-1640 medium (Corning, Corning, NY, USA) containing 10% fetal bovine serum (FBS; Tianhang Biotechnology, Zhejiang, China) at 37°C in a humidified atmosphere containing 5% CO_2_.

### Evaluation of A549 cell proliferation

The Cell Counting Kit-8 (CCK-8) assay was used to assess cell proliferation. Cells in 96-well culture plates (5 × 10^3^ cells/well) were cultured in medium containing 10% FBS overnight (37°C, 5% CO_2_) and then in medium containing various concentrations of human recombinant AIF-1 (rAIF-1; Cusabio, Wuhan, China) for 24, 48, or 72 hours (37°C, 5% CO_2_). Next, 10 μL CCK-8 solution (ReportBio, Shijiazhuang, China) was added to each well, and the culture plate was incubated for 1 hour at 37°C in a humidified atmosphere containing 5% CO_2_. The absorbance at 450 nm was measured using a plate reader (Multiskan FC microplate photometer, Thermo Fisher Scientific, Waltham, MA, USA), and cell proliferation rate was determined.

### Wound healing assay

Cells were seeded in 6-well culture plates (6.0 × 10^5^ cells/well) and grown to 90% confluence. The cell monolayer was scraped using a 200 μL pipette tip, and the medium was changed to medium containing 1% FBS with or without rAIF-1 (400 ng/mL). Cells were observed and photographed at 0, 24, 48 and 72 hours, and wound healing was quantified using ImageJ software (National Institutes of Health, Bethesda, MD, USA).

### Western blot analysis

Collected cells were lysed with radioimmunoprecipitation assay buffer containing protease inhibitor and phosphatase inhibitor (on ice on a shaking platform for 30 min). Then, the cells were homogenized by ultrasonic disruption and centrifuged at 12,000 rpm for 15 minutes, and the supernatant was collected. Proteins were quantified using the BCA Protein Assay Kit (Takara Bio, Kusatsu, Japan). Denatured proteins were separated by 10% sodium dodecyl sulfate-polyacrylamide gel electrophoresis (SDS–PAGE) or 12% SDS-PAGE and transferred to a Hybond membrane (Amersham, UK), which was blocked for 1 hour in 5% BSA in Tris-buffered saline/Tween-20 (TBST). The membrane was incubated with primary antibodies (S1 Table) overnight at 4°C, washed with TBST and incubated with HRP-conjugated IgG (1:5000; Bioss, Beijing, China) for 120 minutes. Immunoreactive complexes were detected using ECL-Plus detection reagents (Santa Cruz Biotechnology, Santa Cruz, CA, USA).

### Real-time polymerase chain reaction (RT-PCR)

RNA was extracted using the Superbrilliant^TM^ 6 min High-quality RNA Extraction kit (Zhongshi Tontru, Tianjin, China). cDNA was synthesized from 1 μg RNA using the FastKing RT Kit (Tiangen, Beijing, China). The oligonucleotide primers were designed according to Genbank (S2 Table). RT-PCR was carried out using the SuperReal PreMix Plus (SYBR Green) kit (Tiangen, Beijing, China) in accordance with the manufacturer’s instructions.

### Quantification of IL-6 and VEGF levels

IL-6 and VEGF levels in culture supernatants were quantified using the Elecsys IL-6 immunoassay kit (Roche, Basel, Switzerland), Cobas e 411 electrochemiluminescence analyzer (Roche, Basel, Switzerland), Vascular Endothelial Growth Factor Assay Kit (Yida Qihang, Beijing, China), and TZD-200S chemiluminescence immunoanalyzer (Tianzhongda Biotech, Xiamen, China).

### Statistical analysis

Data were analyzed using SPSS 25.0 (IBM, Armonk, NY, USA). Normally distributed measurement data were expressed as mean ± standard deviation and were compared between groups using analysis of variance (ANOVA) and Tukey’s post-hoc test. Ranked data were analyzed using the chi-squared test. Survival analyses were performed using the Kaplan-Meier method and log-rank test. For disease-specific survival analysis, the endpoint was death with or from NSCLC [31]. The relationships between the mRNA expressions of different proteins were evaluated using Pearson correlation analyses. *P* < 0.05 was considered statistically significant.

## Results

### Expression of AIF-1 in NSCLC

**Fig 1:**
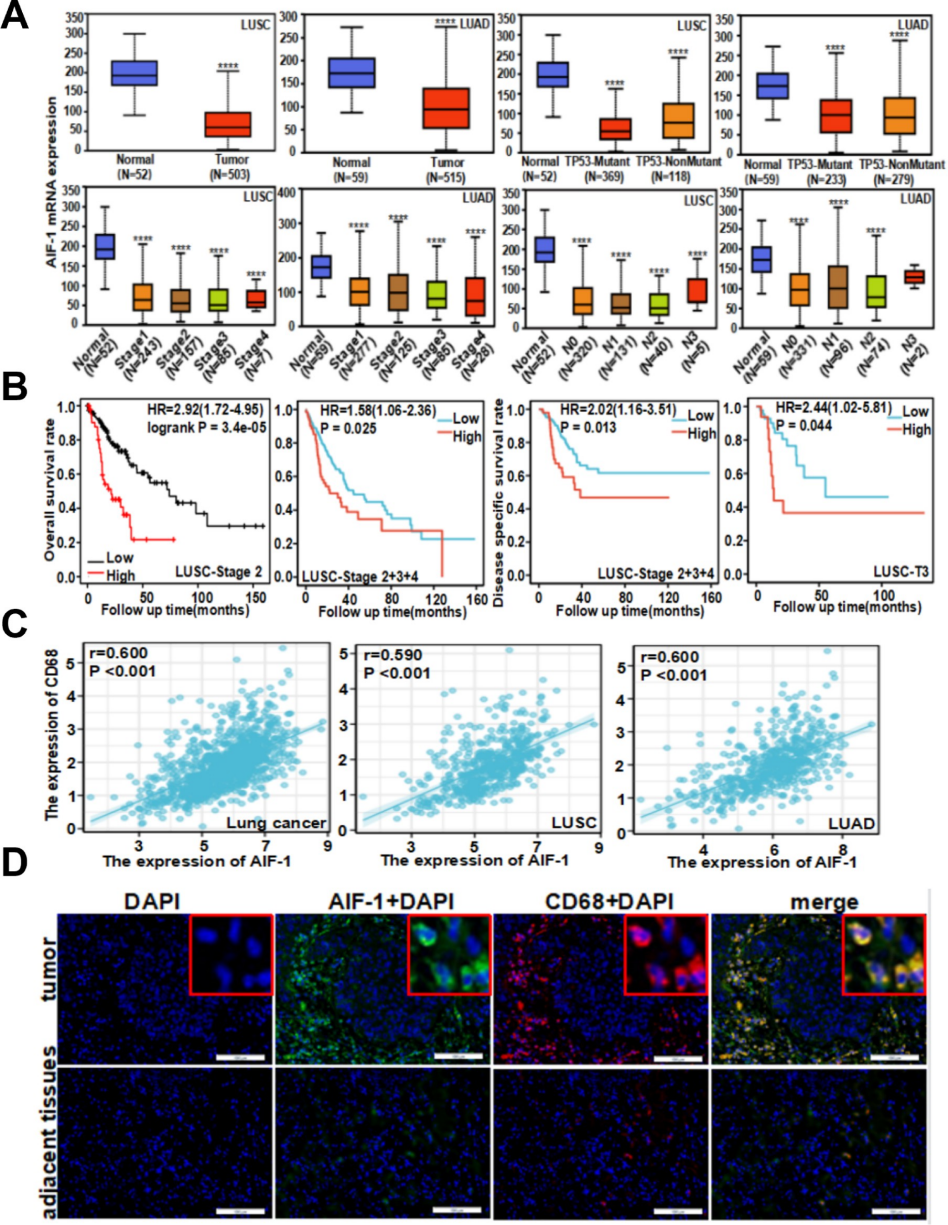
Expression of AIF-1 in NSCLC. (**A**) AIF-1 mRNA expression in NSCLC tissue obtained from TCGA database analyses. LUAD: Lung adenocarcinoma; LUSC: Lung SCC. Boxplots show median, interquartile range and range. **** *P* < 0.0001. (**B**) Kaplan-Meier analyses comparing overall survival (patients with stage II or stage II–IV lung SCC) and disease-specific survival (patients with stage II–IV or stage T3 lung SCC) between those with high AIF-1 expression (High) and those with low AIF-1 expression (Low). HR: Hazard ratio (with 95% confidence interval). (**C**) Pearson correlation analysis of the relation between AIF-1 expression and CD68 expression in patients with lung cancer, lung SCC (LUSC) and lung adenocarcinoma (LUAD). (**D**) Double immunofluorescence staining of NSCLC samples and adjacent tissues. AIF-1 (green) and CD68 (red) were co-localized (yellow) in lung macrophages around nests of tumor tissue. Nuclei are stained blue with DAPI. Scale bar: 100 μm.

NSCLC tissue obtained from the 47 participants exhibited a higher rate of AIF-1 protein expression than paracancer tissue from the same patients (63.3% vs. 41.7%, *P* < 0.05; S3 Table). However, analysis of TGCA database data revealed that AIF-1 mRNA expression was significantly lower in lung adenocarcinoma and lung SCC than in normal tissue irrespective of p53 mutation status, disease stage or nodal stage (*P* < 0.001 vs. normal tissue; Fig 1A). Analyses of bioinformatics data revealed that overall survival rate and disease-specific survival rate were significantly higher in patients with low AIF-1 mRNA expression than in those with high AIF-1 mRNA expression (*P* < 0.05; Fig 1B). Pearson correlation analyses of TGCA data (Fig 1C) showed that AIF-1 expression was significantly positively correlated with CD68 expression (a macrophage marker) in NSCLC (*r* = 0.600, *P* < 0.001), lung SCC (*r* = 0.590, *P* < 0.001) and lung adenocarcinoma (*r* = 0.600, *P* < 0.001). Furthermore, immunofluorescence double-labeling experiments demonstrated co-localization of AIF-1 and CD68 in cells within cancer nests, suggesting that AIF is mainly expressed in the cytoplasm of macrophages infiltrating the tumor tissue (Fig 1D).

### Relationship between AIF-1 expression and the clinicopathological characteristics of NSCLC

Immunostaining of the tissue samples from the 47 participants (S4 Table) revealed that the rate of positive AIF-1 expression was significantly higher for patients with lymph node metastasis (*P* < 0.05 vs. no lymph node metastasis) TNM stage III–IV (*P* < 0.05 vs. TNM stage 0–II), and positivity for Ki67 expression (*P* < 0.05 vs. no Ki67 expression). However, AIF-1 expression was not associated with age, sex, tumor differentiation, tumor type or p53 expression (S4 Table).

### Relationship between AIF-1 expression and the expressions of IL-6 and VEGF in patients with NSCLC

**Fig 2:**
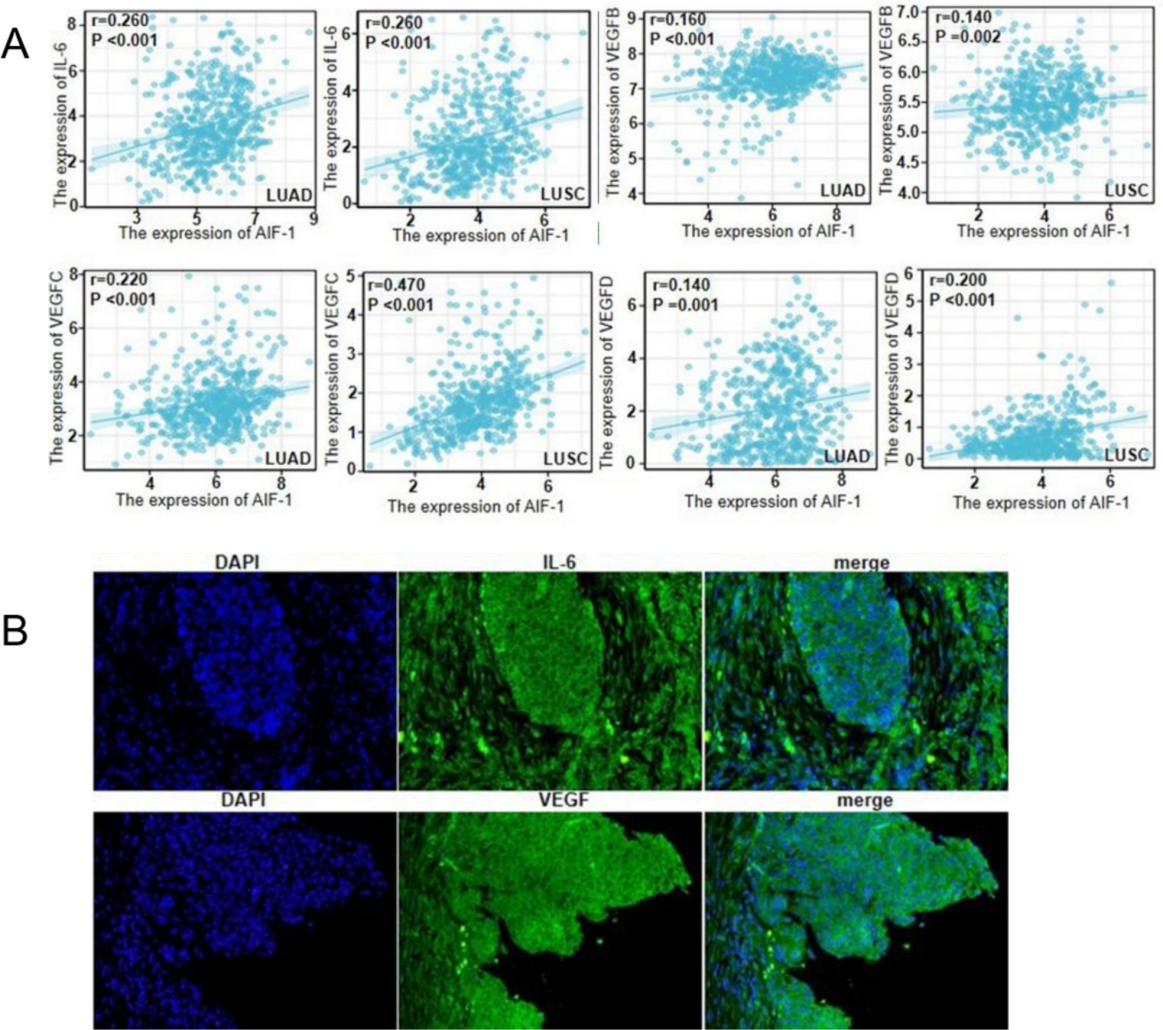
Relationship between AIF-1 expression and the expressions of IL-6 and VEGF in NSCLC tissue. (**A**) Pearson correlation analyses of the relations between AIF-1 expression and the expressions of IL-6, VEGF-B, VEGF-C, and VEGF-D in lung adenocarcinoma tissue (LUAD) and lung SCC tissue (LUSC). Data were obtained from TCGA database. (**B**) Immunofluorescence staining of IL-6 and VEGF in NSCLC samples. IL-6 and VEGF were localized in the cytoplasm and intercellular stroma (green). Nuclei are stained blue with DAPI. Scale bar: 100 μm.

Immunofluorescence staining of NSCLC tissues from the study participants (S4 Table) showed that the rate of positive AIF-1 expression was significantly higher for patients with positivity for IL-6 expression (*P* < 0.01 vs. no IL-6 expression) and positivity for VEGF expression (*P* < 0.05 vs. no VEGF expression). Analysis of TCGA data also showed that AIF-1 expression was significantly positively correlated with the expression of IL-6 (*P* < 0.001), VEGF-B (*P* < 0.01), VEGF-C (*P* < 0.001), and VEGF-D (*P* < 0.001) (Fig 2A). Immunofluorescence staining of NSCLC samples showed that IL-6 and VEGF were localized in the cytoplasm and intercellular stroma (Fig 2B).

### Analysis of pathways associated with AIF-1

**Fig 3:**
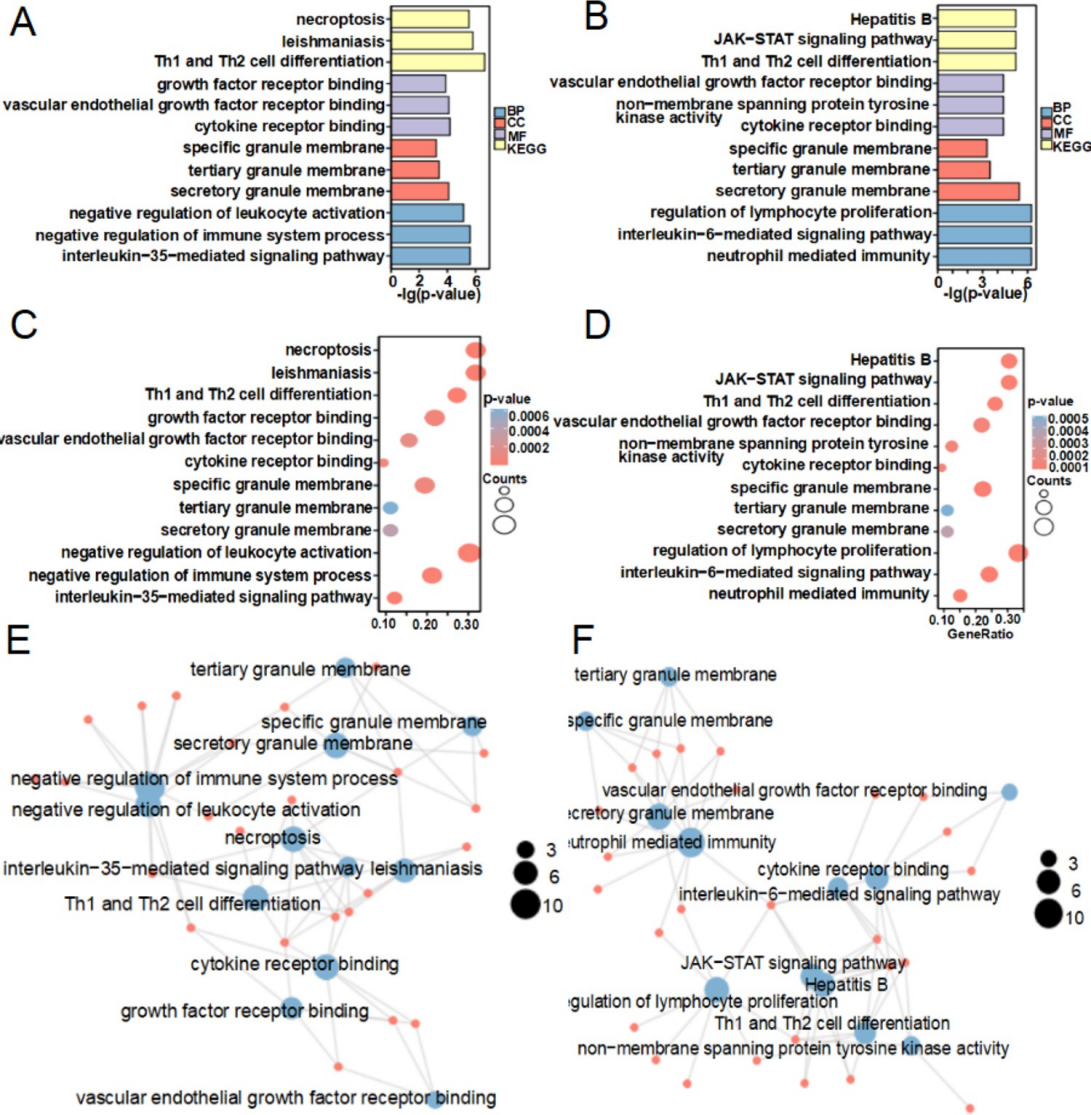
Enrichment analyses of AIF1-related signaling pathways. (**A**) Bar plot showing the enriched terms for lung SCC. (**B**) Bar plot showing the enriched terms for lung adenocarcinoma. (**C**) Dot plot showing the enriched terms for lung SCC. (**D**) Dot plot showing the enriched terms for lung adenocarcinoma. **(E**) Network plot showing the enriched terms for lung SCC. (**F**) Network plot showing the enriched terms for lung adenocarcinoma.

Gene correlation analyses of TCGA data revealed that several genes associated with p38-MAPK and JAK/STAT3 signaling were strongly positively correlated with AIF-1 expression in lung SCC (S5 Table) and lung adenocarcinoma (S6 Table). Gene ontology and KEGG pathway enrichment analyses of AIF-1-related signaling pathways in NSCLC showed that the enriched pathways included necroptosis, leishmaniasis, Th1 and Th2 cell differentiation, growth factor receptor binding, VEGF receptor binding, cytokine receptor binding, specific granule membrane, tertiary granule membrane, secretory granule membrane, negative regulation of leukocyte activation, negative regulation of immune system process, IL-35-mediated signaling pathway, hepatitis B, JAK/STAT signaling pathway, non-membrane spanning protein tyrosine kinase activity, regulation of lymphocyte proliferation, IL-6-mediated signaling pathway, and neutrophil mediated immunity (Fig 3).

### rAIF-1 up-regulated the expressions of IL-6 and VEGF in A549 cells

**Fig 4:**
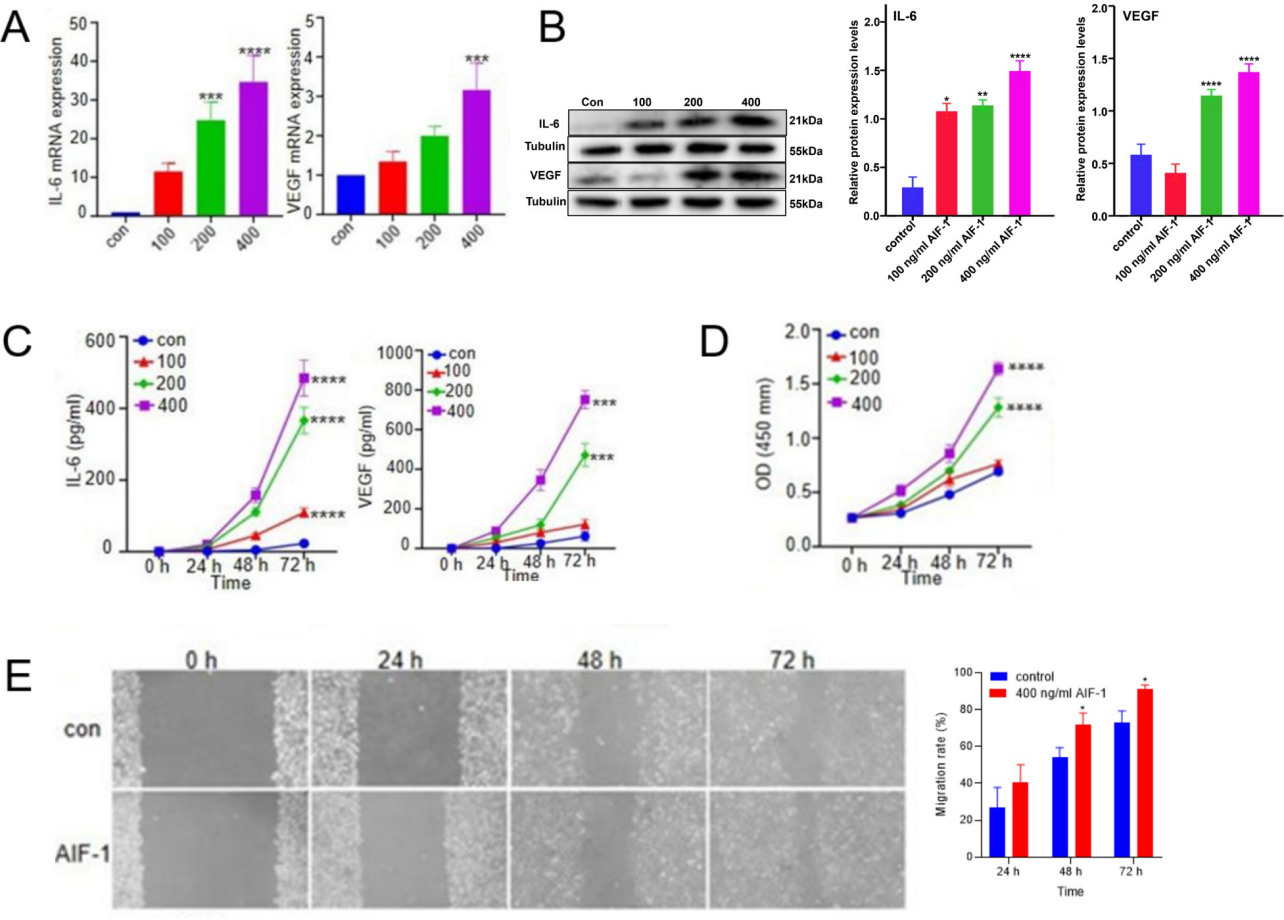
Effects of rAIF-1 on A549 cell proliferation, migration and production of IL-6 and VEGF. (**A**) Concentration-dependent effects of rAIF-1 on the mRNA expressions of IL-6 and VEGF. Data are shown as mean ± SD. *** *P* < 0.001; **** *P* < 0.0001. (**B**) Concentration-dependent effects of rAIF-1 on the protein expressions of IL-6 and VEGF. Data are shown as mean ± SD. * *P* < 0.05; ** *P* < 0.01; **** *P* < 0.0001. (**C**) Culture medium levels of IL-6 and VEGF secreted by A549 cells. Data are shown as mean ± SD. *** *P* < 0.001; **** *P* < 0.0001. (**D**) Viability of A549 cells treated with 100, 200 or 400 ng/mL rAIF-1 (CCK-8 assay). OD: Optical density. Data are shown as mean ± SD. **** *P* < 0.0001. (**E**) Migration of A549 cells treated with 400 ng/mL rAIF-1 (wound healing assay). Data are shown as mean ± SD. * *P* < 0.05.

The possible functional role of AIF-1 in NSCLC was further explored in a human lung cancer cell line. rAIF-1 (100, 200 or 400 ng/mL) caused concentration-dependent increases in the mRNA and protein expressions of IL-6 and VEGF in A549 cells cultured *in vitro* (Fig 4A and 4B). Furthermore, the culture medium levels of IL-6 and VEGF (i.e., the amounts of IL-6 and VEGF secreted by A549 cells) were also increased by rAIF-1 in a concentration-dependent manner (Fig 4C).

### rAIF-1 stimulated the proliferation and migration of A549 cells

CCK-8 assays demonstrated that rAIF-1 (200 or 400 ng/mL) stimulated the proliferation of A549 cells in a concentration-dependent manner (*P* < 0.001 vs. control at 72 hours; Fig 4D). Wound healing assays showed that 400 ng/mL rAIF-1 significantly enhanced the migration of A549 cells (*P* < 0.05 vs. control at 48 hours and 72 hours; Fig 4E).

### rAIF-1 activated p38-MAPK and JAK/STAT3 signaling in A549 cells

**Fig 5:**
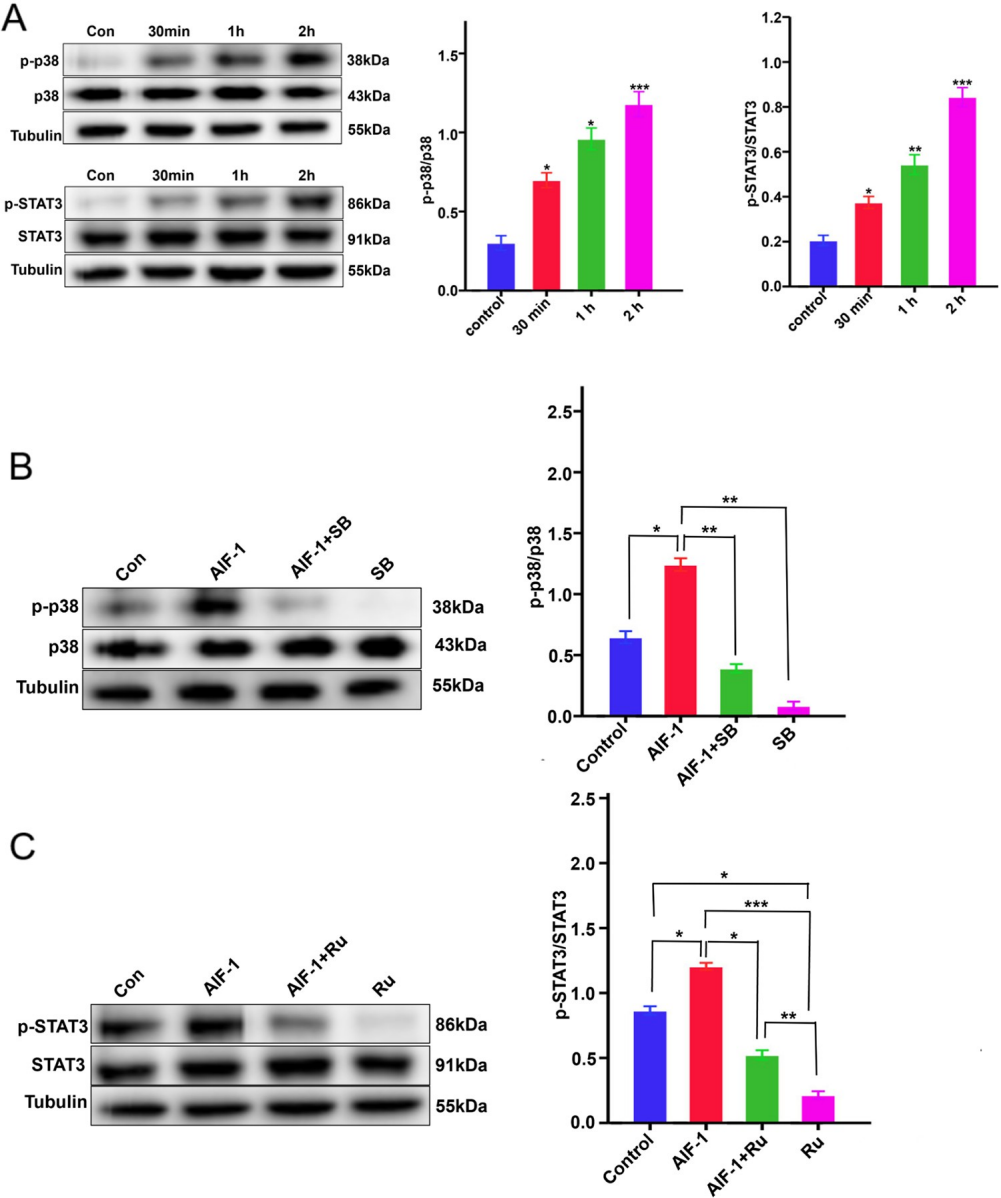
SB203580 and ruxolitinib attenuated the activation of p38-MAPK signaling and JAK/STAT3 signaling by rAIF-1 in A549 cells. (**A**) Left: Representative blots showing the expressions of p-p38, total p38, p-STAT3, total STAT3 and tubulin (internal control). Right: Averaged data showing the effects of 400 ng/mL rAIF-1 on the p-STAT3/ STAT3 ratio and p-p38/p38 ratio. Data are shown as mean ± SD. * *P* < 0.05; ** *P* < 0.01; *** *P* < 0.001. (**B**) SB203580, an inhibitor of p38-MAPK signaling, attenuated the increase in the p-p38/p38 ratio in A549 cells induced by rAIF-1. Data are shown as mean ± SD. * *P* < 0.05; ** *P* < 0.01. (**C**) Ruxolitinib, an inhibitor of JAK/STAT3 signaling, attenuated the elevation in the p-STAT3/STAT3 ratio in rAIF-1-treated A549 cells. Data are shown as mean ± SD. * *P* < 0.05; ** *P* < 0.01; *** *P* < 0.001.

Western blot experiments indicated that treatment of A549 cells with 400 ng/mL rAIF-1 resulted in significant increases in the levels of phosphorylated p38 (p-p38) and p-STAT3 at 30 minutes, 1 hour, and 2 hours (*P* < 0.05 for p-p38 and p-STAT3 at all time points), indicating that rAIF-1 activated p38-MAPK and JAK/STAT3 signaling in these cells (Fig 5A). Furthermore, the up-regulation of p-p38 and p-STAT3 by rAIF-1 was attenuated by SB203580, an inhibitor of p38-MAPK signaling (*P* < 0.01 vs. rAIF-1 alone; Fig 5B), and ruxolitinib, an inhibitor of JAK/STAT3 signaling (*P* < 0.01 vs. rAIF-1 alone; Fig 5C). SB203580 alone had no effect on the p-p38/p38 ratio, implying that there was little or no activation of the p38-MAPK pathway in the absence of rAIF-1 (Fig 5B). By contrast, ruxolitinib alone significantly reduced the p-STAT3/STAT3 ratio, indicating that there was a basal level of JAK/STAT3 signaling in A549 cells (*P* < 0.05 vs. control; Fig 5C).

### The rAIF-1-induced up-regulation of IL-6 and VEGF in A549 cells was associated with activation of p38-MAPK and JAK/STAT3 signaling

**Fig 6:**
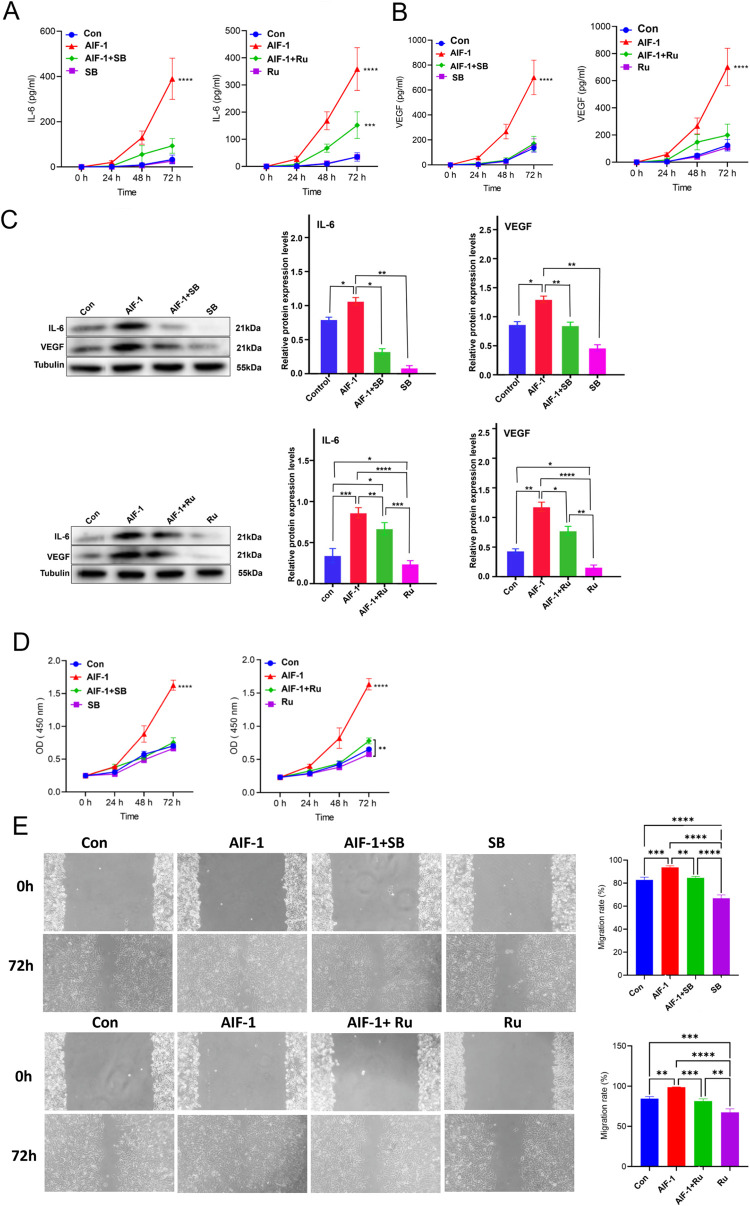
SB203580 and ruxolitinib attenuated the effects of rAIF-1 on A549 cell proliferation, migration, and production of IL-6 and VEGF. (**A**) Culture medium levels of IL-6 secreted by A549 cells. Ru: Ruxolitinib; SB: SB203580. Data are shown as mean ± SD. *** *P* < 0.001; **** *P* < 0.0001. (**B**) Culture medium levels of VEGF secreted by A549 cells. Ru: Ruxolitinib; SB: SB203580. Data are shown as mean ± SD. **** *P* < 0.0001. (**C**) Protein expressions of IL-6 and VEGF. Data are shown as mean ± SD. * *P* < 0.05; ** *P* < 0.01; *** *P* < 0.001; **** *P* < 0.0001. (**D**) Viability of A549 cells determined using the CCK-8 assay. OD: Optical density. Ru: Ruxolitinib; SB: SB203580. Data are shown as mean ± SD. ** *P* < 0.01; **** *P* < 0.0001. (**E**) Migration of A549 cells evaluated using the wound healing assay. Data are shown as mean ± SD. ** *P* < 0.01; *** *P* < 0.001; **** *P* < 0.0001.

In view of the above results, experiments were carried out to determine whether the effects of rAIF-1 on the expressions of IL-6 and VEGF in A549 cells involved p38-MAPK and JAK/STAT3 signaling. IL-6 (Fig 6A) and VEGF (Fig 6B) secreted by rAIF-1-stimulated A549 cells (i.e., culture medium levels of IL-6 and VEGF) were markedly reduced by SB203580 and ruxolitinib. As shown in Fig 6C, the expression of IL-6 protein in rAIF-1-treated A549 cells was significantly decreased by SB203580 (*P* < 0.05 vs. rAIF-1 alone) and ruxolitinib (*P* < 0.01 vs. rAIF-1 alone), and the expression of VEGF protein in rAIF-1-treated A549 cells was also reduced by SB203580 (*P* < 0.01 vs. rAIF-1 alone) and ruxolitinib (*P* < 0.05 vs. rAIF-1 alone). The above findings suggest that rAIF-1 up-regulated the expressions of IL-6 and VEGF in A549 cells by activating the p38-MAPK and JAK/STAT3 signaling pathways.

### Stimulation of A549 cell proliferation and migration by rAIF-1 is associated with activation of p38-MAPK and JAK/STAT3 signaling

Finally, we investigated whether p38-MAPK and JAK/STAT3 signaling are involved in the effects of rAIF-1 on A549 cell proliferation and migration. CCK-8 assays revealed that the stimulation of A549 cell proliferation by rAIF-1 was markedly attenuated by SB203580 and ruxolitinib (Fig 6D). Furthermore, the wound healing assay demonstrated that the migration of rAIF-1-treated A549 cells was inhibited by SB203580 (*P* < 0.01 vs. rAIF-1 alone; Fig 6E) and ruxolitinib (*P* < 0.001 vs. rAIF-1 alone; Fig 6E).

## Discussion

In this study, we demonstrated that AIF-1 protein expression was significantly upregulated in human NSCLC tissue compared with that in paired paracancer tissue, positively associated with tumor stage and metastasis, and negatively correlated with the outcomes of patients. AIF-1 was mainly expressed in the cytoplasm of macrophages infiltrating the tumor tissue and positively correlated with IL-6 and VEGF expression in both clinical NSCLC samples and TCGA NSCLC samples. Bioinformatics analysis revealed that AIF-1 was highly associated with immune and inflammation-related signaling pathways including p38-MAPK and JAK/STAT3 signaling. In vitro study verified that rAIF-1 upregulated IL-6 and VEGF expression and activated p38-MAPK and JAK/STAT3 signaling in A549 cells, accompanied by enhancement of cell proliferation and migration. Importantly, these effects were abolished by p38-MAPK or JAK/STAT3 signaling inhibitor. Taken together, our results suggest that AIF-1 contributes to the growth and inflammatory microenvironment development of NSCLC through p38-MAPK and JAK/STAT3 signaling.

In the present study, immunofluorescence staining of NSCLC samples showed that AIF-1 was mainly located in the cytoplasm of macrophages that infiltrated the cancerous tissue, and AIF-1 expression was higher in tumor tissue than in paracancer tissue. Our results align closely with previous studies of AIF-1 expression in human and rat macrophages infiltrating glioma tissue [32] and mouse macrophages in a model of breast cancer metastasis [33]. We also found that the AIF-1 expression level in NSCLC tissue was positively associated with malignant characteristics such as lymph node metastasis, and TNM stage. This finding is in line with previous data obtained for hepatocellular carcinoma [34]. Furthermore, lymph node involvement was associated with an elevated serum level of AIF-1 in patients with cervical cancer [35], supporting the proposal that AIF-1 may be a marker of more aggressive tumor behavior. Jia et al. reported that AIF-1 promoted HepG2 cell proliferation via insulin-like growth factor-1 receptor signaling [29], while Li et al. found that AIF-1 enhanced the migration of MDA-MB-231 and MCF-7 cells via the up-regulation of tumor necrosis factor-alpha (TNF-α) and p38-MAPK signaling [36]. However, according to our TCGA database analyses, AIF-1 mRNA expression was lower in NSCLC tissue than in normal tissue. A possible reason for this apparent discrepancy is that AIF-1 is mainly expressed in infiltrating macrophages around cancer tissues, whereas TCGA-based analyses considered AIF-1 expression within the cancer tissue itself. This is supported by our findings showing that AIF-1 expression was significantly, positively correlated with CD68 expression in lung cancer and co-localized with CD68^+^ macrophages. CD68 is a pan-macrophage marker that has been extensively used to identify both M1 and M2 tumor-associated macrophages (TAMs) when combined with other macrophage markers[37]. A higher degree of CD68^+^ macrophage infiltration is associated with poor prognosis of different types of cancer, including NSCLC [37–39]. AIF-1 has been found to co-localize with CD68^+^ macrophages in human atherosclerotic coronary arteries, renal interstitium, and synovial membrane, being considered a marker of activated macrophages and endowed with prognostic value for inflammation-related diseases including cancer [38,40,41]. Taken together, our results indicate that AIF-1 is expressed in macrophages that infiltrate NSCLC tissue and that higher AIF-1 expression is associated with more aggressive behavior of cancer. Further research is needed to establish whether AIF-1 might be useful as a biomarker of aggressive tumor behavior in patients with NSCLC.

A549 cells cultured *in vitro* were used to explore the influence of AIF-1 on tumor cell proliferation and migration. We found that rAIF-1 increased the proliferation and migration of A549 cells. In agreement with our observations, Sommerville et al. showed that adenoviral transduction of AIF-1 enhanced the proliferation and migration of rat primary VSMCs and that knockdown of AIF-1 by RNA interference inhibited the proliferation and migration of rat VSMCs [42]. Jia et al. observed that overexpression of AIF-1 up-regulated the migration and proliferation of human endothelial cells by increasing basic fibroblast growth factor [43]. Hence, our results and those of others imply that AIF-1 might promote tumor progression by enhancing proliferation and migration.

Tumor-associated inflammation was first proposed by Rudolf Virchow in the 19th century [44] and is now believed to be an important driver of malignant progression [45]. Cancer-related inflammation appears before tumors such as colitis or hepatitis [46], and many inflammatory cytokines, growth factors, and angiogenic proteins are involved in this process. Under certain conditions, inflammation can result in the accumulation of carcinogenic mutations [47], and in turn, carcinogenic mutations in cells can promote tumor-related inflammation. Chemokines and cytokines produced in response to gastrointestinal and lung cancers can alter tissue structure and oxygen partial pressure and impair barrier integrity, which facilitates the translocation of microbial products. AIF-1 plays a crucial role in the immune system and promotes the secretion of inflammatory factors [48]. The present study found that rAIF-1 increased the production of IL-6 and VEGF by cultured A549 cells. VEGF is a chemoattractant for TAMs. Studies have shown that M2-polarized TAMs can secret VEGF and other pro-angiogenic and tumor-inducing chemokines, forming a positive feedback loop in tumor angiogenesis [49,50]. In addition, IL-6 can induce macrophage infiltration in NSCLC, which in turn promtes cancer cell invasion and angiogenesis in lung cancer [51,52]. Thus, the pro-inflammatory effects of AIF-1 may contribute to the promotion of aggressive tumor behavior.

AIF-1 has been reported to activate several signaling pathways including those of nuclear factor-kappa-B (NF-kB), p38-MAPK, and mammalian target of rapamycin (mTOR) [53–55]. Our bioinformatics analyses connected AIF-1 to the activation of JAK/STAT signaling, and our experiments in A549 cells revealed that rAIF-1 activated p38-MAPK signaling and JAK/STAT signaling. Notably, pharmacological inhibition of p38-MAPK signaling or JAK/STAT signaling attenuated the rAIF-1-induced enhancement of A549 cell proliferation, migration, and production of IL-6 and VEGF.

In conclusion, our results indicate that AIF-1 promotes the proliferation, migration, and production of IL-6 and VEGF by cultured A549 cells via activation of p38-MAPK and JAK/STAT signaling. Furthermore, AIF-1 expression in NSCLC tissue was positively associated with aggressive tumor behavior as evidenced by lymph node metastasis, and advanced TNM stage. Our findings suggest that AIF-1 might play an important role in the development and progression of lung cancer. Additional research is merited to determine whether AIF-1 might be a useful biomarker of aggressive tumor behavior in patients with NSCLC.

## Supporting information

S1 Checklist STROBE statement—Checklist of items that should be included in reports of observational studies. (DOCX)

S1 Fig AIF-1 mRNA expression in NSCLC tissue obtained from TCGA database analyses. LUAD: Lung adenocarcinoma; LUSC: Lung SCC. Boxplots show median, interquartile range and range. ** *P* < 0.01; *** *P* < 0.001; **** *P* < 0.0001.
(TIF)

S1 Table Antibodies used for Western blot, immunohistochemistry and immunofluorescence Staining. (DOCX)

S2 Table The oligonucleotide primer sequences and product size for PCR amplification. (DOCX)

S3 Table AIF-1 expression of NSCLC and adjacent tissue tissues. (DOCX)

S4 Table Relationship between AIF-1, clinicopathological features,IL-6 and VEGF in NSCLC. (DOCX)

S5 Table AIF-1-related molecules in lung squamous cell carcinoma. (DOCX)

S6 Table AIF-1-related molecules in lung adenocarcinoma. (DOCX)
