# Supplementary figures and images for: AIF-1, a potential biomarker of aggressive tumor behavior in patients with non-small cell lung cancer
Source: PLoS One. 2022 Dec 15;17(12):e0279211. doi: 10.1371/journal.pone.0279211 (PMC9754194; doi:10.1371/journal.pone.0279211)

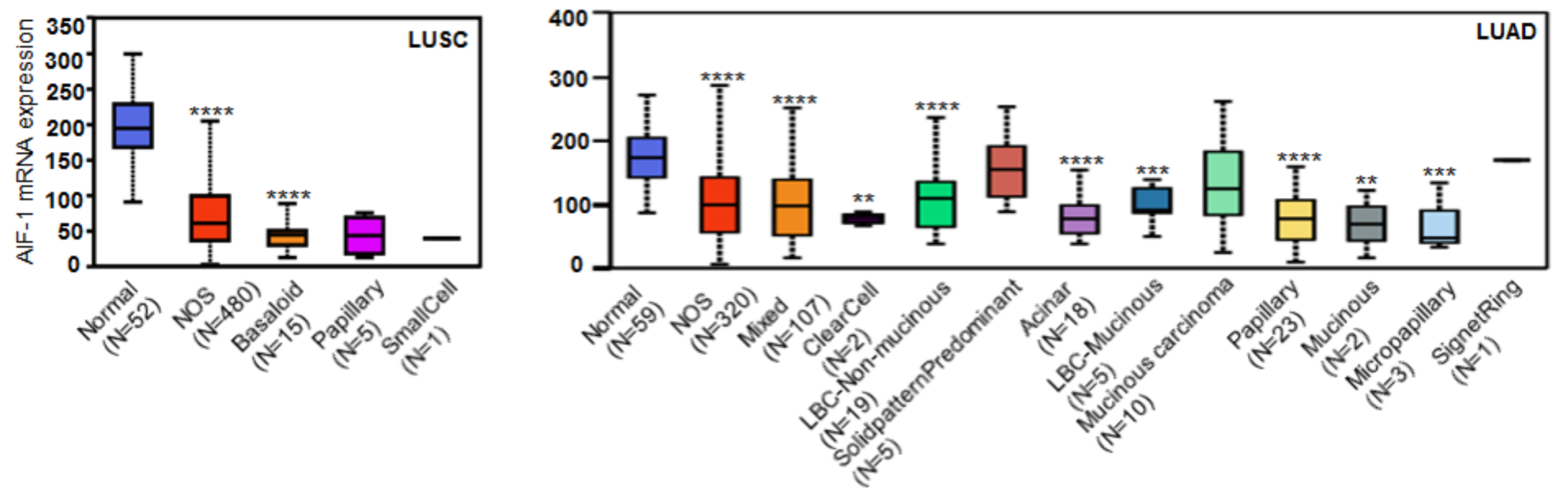

Supplement: S1 Fig — LUAD: Lung adenocarcinoma; LUSC: Lung SCC. Boxplots show median, interquartile range and range. ** P < 0.01; *** P < 0.001; **** P < 0.0001. (TIF) [file pone.0279211.s002.tif]
