# Supplementary material for: AIF-1, a potential biomarker of aggressive tumor behavior in patients with non-small cell lung cancer
Source: PLoS One. 2022 Dec 15;17(12):e0279211. doi: 10.1371/journal.pone.0279211 (PMC9754194; doi:10.1371/journal.pone.0279211)
Supplement: S1 Table — (DOCX) [file pone.0279211.s003.docx]

**S1 Table.** Antibodies used for Western blot, immunohistochemistry and immunofluorescence Staining

| **Name** | **Dilution** | **Source** | **Company** |
| --- | --- | --- | --- |
| **α**-tubulin | 1:1000 | Mouse | Santa Cruz Biotechnology |
| CD68 | 1:100 | Mouse | Santa Cruz Biotechnology |
| AIF-1 | 1:100 | Rabbit | Wanleibio |
| VEGF | 1:1000 | Rabbit | Wanleibio |
| IL-6 | 1:1000 | Rabbit | Wanleibio |
| P38 | 1:500 | Rabbit | Wanleibio |
| P-P38 | 1:500 | Rabbit | Wanleibio |
| STAT3 | 1:500 | Rabbit | Wanleibio |
| P-STAT3 | 1:1000 | Rabbit | Wanleibio |
| Ki67 | 1:1 | Mouse | ZSGB-BIO |
| P53 | 1:1 | Mouse | ZSGB-BIO |
