# Supplementary material for: AIF-1, a potential biomarker of aggressive tumor behavior in patients with non-small cell lung cancer
Source: PLoS One. 2022 Dec 15;17(12):e0279211. doi: 10.1371/journal.pone.0279211 (PMC9754194; doi:10.1371/journal.pone.0279211)
Supplement: S2 Table — (DOCX) [file pone.0279211.s004.docx]

**S2 Table.** The oligonucleotide primer sequences and product size for PCR amplification

| **Name** | **Primer’s sequence** | **Product size (bp)** |
| --- | --- | --- |
| GAPDH | F: 5’-GTCTCCTCTGACTTCAACAGCG-3’ | 131 |
|  | R: 5’-ACCACCCTGTTGCTGTAGCCAA- 3’ |  |
| IL-6 | F: 5’-AGACAGCCACTCACCTCTTCAG-3’ | 132 |
|  | R: 5’-TTCTGCCAGTGCCTCTTTGCTG- 3’ |  |
| VEGF | F: 5’-TTTCTGCTGTCTTGGGTGCA-3’ | 145 |
|  | R: 5’-AGCTGCGCTGATAGACATCC- 3’ |  |
| MIP-1α | F: 5’-ACTTTGAGACGAGCAGCCAGTG-3’ | 101 |
|  | R: 5’-TTTCTGGACCCACTCCTCACTG- 3’ |  |
| MIP-1β | F: 5’-GCTTCCTCGCAACTTTGTGGTAG -3’ | 140 |
|  | R: 5’- GGTCATACACGTACTCCTGGAC - 3’ |  |
| MCP-1 | F: 5’-AGAATCACCAGCAGCAAGTGTCC -3’ | 98 |
|  | R: 5’- TCCTGAACCCACTTCTGCTTGG- 3’ |  |
| IL-8 | F: 5’-GAGAGTGATTGAGAGTGGACCAC -3’ | 112 |
|  | R: 5’-CACAACCCTCTGCACCCAGTTT - 3’ |  |
| IL-1α | F: 5’-TGTATGTGACTGCCCAAGATGAAG-3’ | 96 |
|  | R: 5’- AGAGGAGGTTGGTCTCACTACC- 3’ |  |
| TNF-α | F: 5’-CTCTTCTGCCTGCTGCACTTTG -3’ | 135 |
|  | R: 5’- ATGGGCTACAGGCTTGTCACTC- 3’ |  |
