# Supplementary material for: AIF-1, a potential biomarker of aggressive tumor behavior in patients with non-small cell lung cancer
Source: PLoS One. 2022 Dec 15;17(12):e0279211. doi: 10.1371/journal.pone.0279211 (PMC9754194; doi:10.1371/journal.pone.0279211)
Supplement: S3 Table — (DOCX) [file pone.0279211.s005.docx]

**S3 Table**. AIF-1 expression of NSCLC and adjacent tissue tissues

| Groups | n |  | AIF-1 | | PR (%) | X^2^ | P value |
| --- | --- | --- | --- | --- | --- | --- | --- |
|  |  |  | low | high |  |  |  |
| NSCLC tissues | 47 |  | 17 | 30 | 63.8 | 5.158 | 0.023 |
| Paracancerous tissues | 47 |  | 28 | 19 | 40.4 |  |  |
