# Supplementary material for: AIF-1, a potential biomarker of aggressive tumor behavior in patients with non-small cell lung cancer
Source: PLoS One. 2022 Dec 15;17(12):e0279211. doi: 10.1371/journal.pone.0279211 (PMC9754194; doi:10.1371/journal.pone.0279211)
Supplement: S4 Table — (DOCX) [file pone.0279211.s006.docx]

**S4 Table**. Relationship between AIF-1, clinicopathological features, IL-6, and VEGF in NSCLC

| Clinicopathological features | AIF-1 high group (n = 30) | AIF-1 low group (n = 17) | *X*^2^ | P value |
| --- | --- | --- | --- | --- |
| Lymph node metastasis |  |  | 4.561 | 0.033 |
| − | 20 (66.7) | 16 (94.1) |  |  |
| + | 10 (33.3) | 1 (5.9) |  |  |
| TNM stage |  |  | 6.660 | 0.036 |
| I | 14 (46.7) | 14 (82.4) |  |  |
| II | 7 (23.3) | 2 (11.8) |  |  |
| III | 9 (30.0) | 1 (5.9) |  |  |
| Differentiation |  |  | 1.075 | 0.584 |
| Well-differentiated | 8 (26.7) | 7 (41.2) |  |  |
| Moderately differentiated | 16 (53.3) | 7 (41.2) |  |  |
| Poorly differentiated | 6 (20.0) | 3 (17.6) |  |  |
| Tissue type |  |  | 1.311 | 0.252 |
| Adenocarcinoma | 18 (60.0) | 13 (76.5) |  |  |
| Squamous cell carcinoma | 12 (40.0) | 4 (23.5) |  |  |
| Ki67 |  |  | 8.296 | 0.004 |
| − | 2 (6.7) | 8 (47.1) |  |  |
| + | 28 (93.3) | 9 (52.9) |  |  |
| p53 |  |  | 1.882 | 0.170 |
| − | 15 (50.0) | 12 (70.6) |  |  |
| + | 15 (50.0) | 5 (29.4) |  |  |
| IL-6 |  |  | 24.846 | <0.001 |
| − | 0 (0.0) | 12 (70.6) |  |  |
| + | 30 (100.0) | 5 (29.4) |  |  |
| VEGF |  |  | 18.332 | <0.001 |
| − | 3 (10.0) | 12 (70.6) |  |  |
| + | 27 (90.0) | 5 (29.4) |  |  |
