# Supplementary material for: AIF-1, a potential biomarker of aggressive tumor behavior in patients with non-small cell lung cancer
Source: PLoS One. 2022 Dec 15;17(12):e0279211. doi: 10.1371/journal.pone.0279211 (PMC9754194; doi:10.1371/journal.pone.0279211)
Supplement: S5 Table — (DOCX) [file pone.0279211.s007.docx]

**S5 Table.** AIF-1-related molecules in lung squamous cell carcinoma

| Names | Pearson correlation coefficient | P value |
| --- | --- | --- |
| FCER1G | 0.945 | <0.001 |
| TNFAIP8L2 | 0.936 | <0.001 |
| MS4A6A | 0.930 | <0.001 |
| TYROBP | 0.929 | <0.001 |
| LST1 | 0.929 | <0.001 |
| HAVCR2 | 0.928 | <0.001 |
| C2QA | 0.918 | <0.001 |
| MNDA | 0.916 | <0.001 |
| GMFG | 0.916 | <0.001 |
| LAPTM5 | 0.915 | <0.001 |
| LAIR1 | 0.913 | <0.001 |
| CD53 | 0.910 | <0.001 |
| C1QB | 0.906 | <0.001 |
| C1QC | 0.904 | <0.001 |
| RNASE6 | 0.904 | <0.001 |
| CD300LF | 0.903 | <0.001 |
| SLC7A7 | 0.899 | <0.001 |
| CYBB | 0.897 | <0.001 |
| GIMAP4 | 0.897 | <0.001 |
| IGSF6 | 0.896 | <0.001 |
| HLA-DRA | 0.896 | <0.001 |
| HLA-DMB | 0.896 | <0.001 |
| GPR65 | 0.895 | <0.001 |
| SPI1 | 0.892 | <0.001 |
| CD68 | 0.562 | <0.001 |
| IL6 | 0.249 | <0.001 |
| C1orf162 | 0.888 | <0.001 |
| C3AR1 | 0.887 | <0.001 |
| VEGFC | 0.411 | <0.001 |
| VEGFD | 0.249 | <0.001 |
| GTF2IRD1 | -0.477 | <0.001 |
| PACSIN3 | -0.475 | <0.001 |
| JUP | -0.458 | <0.001 |
| IQANK1 | -0.451 | <0.001 |
| ZNF74 | -0.445 | <0.001 |
| FANCE | -0.444 | <0.001 |
| C6orf136 | -0.439 | <0.001 |
| DGUOK-AS1 | -0.435 | <0.001 |
| PLEKHH3 | -0.434 | <0.001 |
| DDR1 | -0.433 | <0.001 |
| ESS2 | -0.433 | <0.001 |
| KAT2A | -0.431 | <0.001 |
| FAM83H | -0.431 | <0.001 |
| MTA1 | -0.428 | <0.001 |
| FBXL19 | -0.427 | <0.001 |
| SLC6A8 | -0.426 | <0.001 |
| SREBF2-AS1 | -0.425 | <0.001 |
| TTLL12 | -0.425 | <0.001 |
| PRICKLE3 | -0.422 | <0.001 |
| TNK1 | -0.422 | <0.001 |
| PPP1R27 | -0.420 | <0.001 |
| SLC25A1 | -0.420 | <0.001 |
| MZT2A | -0.419 | <0.001 |
| AP001453.3 | -0.418 | <0.001 |
| PCAT6 | -0.417 | <0.001 |
| AC004471.2 | -0.417 | <0.001 |
| OTX1 | -0.415 | <0.001 |
| PTCD1 | -0.415 | <0.001 |
| MORC2 | -0.415 | <0.001 |
| MZT2B | -0.414 | <0.001 |
