# Supplementary material for: AIF-1, a potential biomarker of aggressive tumor behavior in patients with non-small cell lung cancer
Source: PLoS One. 2022 Dec 15;17(12):e0279211. doi: 10.1371/journal.pone.0279211 (PMC9754194; doi:10.1371/journal.pone.0279211)
Supplement: S6 Table — (DOCX) [file pone.0279211.s008.docx]

**S6 Table.** AIF-1-related molecules in lung adenocarcinoma.

| Names | Pearson correlation coefficient | P value |
| --- | --- | --- |
| TNFAIP8L2 | 0.929 | <0.001 |
| FCER1G | 0.925 | <0.001 |
| LST1 | 0.918 | <0.001 |
| HAVCR2 | 0.914 | <0.001 |
| LAPTM5 | 0.909 | <0.001 |
| MS4A6A | 0.899 | <0.001 |
| LAIR1 | 0.899 | <0.001 |
| CD86 | 0.893 | <0.001 |
| MNDA | 0.892 | <0.001 |
| TYROBP | 0.891 | <0.001 |
| GMFG | 0.882 | <0.001 |
| CD33 | 0.881 | <0.001 |
| C1QA | 0.881 | <0.001 |
| SPI1 | 0.877 | <0.001 |
| C1QC | 0.876 | <0.001 |
| C1QB | 0.875 | <0.001 |
| HCK | 0.875 | <0.001 |
| DOK2 | 0.875 | <0.001 |
| LY86 | 0.874 | <0.001 |
| LRRC25 | 0.873 | <0.001 |
| C3AR1 | 0.873 | <0.001 |
| CD53 | 0.872 | <0.001 |
| SIGLEC9 | 0.869 | <0.001 |
| C1orf162 | 0.869 | <0.001 |
| CD68 | 0.570 | <0.001 |
| IL6 | 0.268 | <0.001 |
| CD4 | 0.869 | <0.001 |
| VEGFB | 0.230 | <0.001 |
| VEGFC | 0.197 | <0.001 |
| VEGFD | 0.156 | <0.001 |
| G2CD4D | -0.440 | <0.001 |
| KIAA1549 | -0.435 | <0.001 |
| AC026368.1 | -0.419 | <0.001 |
| AL512413.1 | -0.416 | <0.001 |
| ICA1 | -0.410 | <0.001 |
| AL355987.4 | -0.402 | <0.001 |
| OVOL2 | -0.388 | <0.001 |
| AC005237.1 | -0.381 | <0.001 |
| CCDC183 | -0.377 | <0.001 |
| EYS | -0.375 | <0.001 |
| INHA | -0.372 | <0.001 |
| HYKK | -0.372 | <0.001 |
| SPIRE2 | -0.369 | <0.001 |
| AC069366.1 | -0.366 | <0.001 |
| AL021154.1 | -0.365 | <0.001 |
| TACC2 | -0.365 | <0.001 |
| C1orf56 | -0.364 | <0.001 |
| AC139887.2 | -0.363 | <0.001 |
| AC093585.1 | -0.362 | <0.001 |
| AP006248.2 | -0.361 | <0.001 |
| AC090541.1 | -0.361 | <0.001 |
| AC107068.1 | -0.361 | <0.001 |
| AL355802.3 | -0.358 | <0.001 |
| AC084262.1 | -0.358 | <0.001 |
| GAN | -0.357 | <0.001 |
| IRS2 | -0.354 | <0.001 |
| CABYR | -0.353 | <0.001 |
| AC079384.1 | -0.353 | <0.001 |
| PPARGC1A | -0.352 | <0.001 |
| AP005899.1 | -0.349 | <0.001 |
